# Economic Role of Population Density during Pandemics—A Comparative Analysis of Saudi Arabia and China

**DOI:** 10.3390/ijerph18084318

**Published:** 2021-04-19

**Authors:** Nadia Yusuf, Lamia Saud Shesha

**Affiliations:** Economics Department, King Abdulaziz University, Jeddah 21551, Saudi Arabia; lshisha@kau.edu.sa

**Keywords:** Covid-19, population density, Covid-19 mortality, economic recovery, population reduction, China, Saudi Arabia, Henry Kissinger

## Abstract

As a novel infection with relatively high contagiousness, the coronavirus disease emerged as the most pertinent threat to the global community in the twenty-first century. Due to Covid-19’s severe economic impacts, the establishment of reliable determining factors can help to alleviate future pandemics. While a population density is often cited as a major determinant of infectious cases and mortality rates, there are both proponents and opponents to this claim. In this framework, the study seeks to assess the role of population density as a predictor of Covid-19 cases and deaths in Saudi Arabia and China during the Covid-19 pandemic. With high infectivity and mortality being a definitive characteristic of overpopulated regions, the authors propose that Henry Kissinger’s population reduction theory can be applied as a control measure to control future pandemics and alleviate social concerns. If high-density Chinese regions are more susceptible to Covid-19 than low-density Saudi cities, the authors argue that Neo-Malthusian models can be used as a basis for reducing the impacts of the coronavirus disease on the economic growth in countries with low population density. However, the performed correlation analysis and simple linear regression produced controversial results with no clear connection between the three studied variables. By assessing population density as a determinant of health crises associated with multiple socio-economic threats and epidemiological concerns, the authors seek to reinvigorate the scholarly interest in Neo-Malthusian models as a long-term solution intended to mitigate future disasters. The authors recommend that future studies should explore additional confounding factors influencing the course and severity of infectious diseases in states with different population densities.

## 1. Introduction

A novel infection with high contagiousness, the coronavirus disease (Covid-19) emerged as the most pertinent threat to the global community in the twenty-first century. With a variety of transmission routes underlying the initial spread of the virus, globalization proved to be one of the key factors responsible for the unprecedented rise of infection cases across the world. The lack of evidence-based practice and empirical knowledge on the topic contributed to the ineffectiveness of certain preventive measures and delayed response in most states. As the Covid-19 pandemic paralyzed health systems and impaired national economies, most scholars focused on the short-term prospects of the encountered epidemiological crisis [1]. Seeking to mitigate anticipated economic consequences and ensure recovery of numerous industries, both governments and experts prioritized immediate solutions such as economic stimuli and mass vaccination. However, the importance of overpopulation, with high population density being one of its key functions, as a confounding factor of the pandemic’s severity was largely overlooked. In this framework, the current research aims to evaluate the significance of population density as a determinant for high infection rates and mortality leading to the consequent economic slowdown in overpopulated countries. Furthermore, the authors analyze the validity of Neo-Malthusian models informed by Henry Kissinger’s population control theory under the outlined circumstances by comparing the development of the Covid-19 situation in Saudi Arabia and China.

Emerging in late 2019, the novel coronavirus infection affected the entirety of the world’s population to a different degree. For the purposes of convenience and simplicity, both the infectious disease and the associated viral pathogen are addressed with the acronym Covid-19. With the first cases of Covid-19 being recorded in China’s Hubei province, the severity of the acute respiratory syndrome caused by the SARS-CoV-2 strain of coronaviruses was not immediately apparent [2]. Recognizing the alarming incidence of new outbreaks outside of China, WHO promptly classified the evolving epidemiologic situation as a pandemic. Throughout spring and summer 2020, Covid-19 engulfed most sovereign states without distinction by their development rates, economic prosperity, or population size [3]. By July, the pandemic’s epicenter shifted from its place of origin in China to countries located in Europe and the Americas. While several observers reported more than 14 million global cases and 607,746 deaths due to Covid-19 on 18 July, total Covid-19 cases exceeded 53 million and deaths reached 1.3 million by mid-November 2020 [4]. At the date of the research, the states with the highest recorded numbers of confirmed Covid-19 cases and deaths are the US, Brazil, and India. As the global number of cases exceeds 85 million, the number of fatal outcomes caused by the disease approaches 1.8 million.

Considering that countries at different stages of economic development have been reporting hundreds of thousands of Covid-19 cases and thousands of fatal outcomes on a daily basis, the situation is unlikely to be completely resolved in the upcoming months and years. By the time medical professionals started to identify instances of human-to-human transmission outside of China, the spread of Covid-19 was already out of control [5]. The collective outcome of the pandemic is not limited to immediate health concerns and deaths due to the fact that coronavirus’s impacts encompass numerous spheres of life. Specifically, the implementation of social distancing and other measures aimed to reduce infection rates resulted in social disruptions and economic disturbances on a global scale [6]. The former became especially evident in regions with high population density as local authorities mandated shutdowns of entire industries while severely limiting citizens’ ability to travel, work, and communicate [7]. Unsurprisingly, border closures and lockdowns amounted to a near-complete cessation of commercial and industrial activities in many regions. The resulting economic downturn can be adequately gauged by sharp declines in GDP growth rates as well as the shrinkage of previously robust markets [8]. 

As evident from the presented overview, the severity of detrimental impacts associated with Covid-19 necessitates the development of comprehensive long-term solutions that would help in mitigating socio-economic consequences of future epidemiologic crises. To this end, we performed a comparative analysis of Saudi Arabia and China, two non-democratic countries that successfully curtailed the spread of the coronavirus disease thanks to strict lockdown measures. The choice is further justified by the contrast in their respective population densities—the average for Saudi Arabia is 15.6 inhabitants per sq. km, and for China it is 148 inhabitants per sq. km. Although population density is an established demographic parameter, we approached the investigation from the socio-economic standpoint by assessing the predictive value of population density for a country’s ability to cope with the pandemic [1,4]. Being intrinsically connected with urbanization rates and the economic growth, density levels could have a substantial impact on the country’s capacity for economic recovery. The understanding of socio-economic and epidemiological consequences of overpopulation is integral for determining a long-term solution informed by population reduction models [8]. Accordingly, the study’s contribution to the academia emerges from the assessment of population density as a determinant of the severity of health crises such as the Covid-19 pandemic. While overpopulation is associated with multiple socio-economic threats and epidemiological concerns, we seek to reinvigorate the scholarly interest in Neo-Malthusian models as a long-term solution intended to mitigate future disasters and health crises.

The current study begins with a literature review focusing on the development of the Covid-19 pandemic in Saudi Arabia and China which includes a detailed overview of the epidemiological, political, and economic circumstances in both countries. The subsequent subsection provides a background for using population density as a determinant of the epidemiological situations that can further inform the adoption of a Neo-Malthusian model to address further pandemics. Following the Methodology section that delineates the used quantitative techniques as well as the conceptual framework for qualitative analysis, the research continues with the Results and Discussion section. In the Conclusion section, the authors briefly outline the key findings of the study by reiterating the findings and commenting on the significance of the results in the context of the posed research question. 

## 2. Literature Review

### 2.1. The Course of the Covid-19 Pandemic in Saudi Arabia

Centered on the Arabian Peninsula, The Kingdom of Saudi Arabia is one of the largest and most economically prosperous Middle Eastern states. The population of more than 34 million is unevenly spread across the vast area of 2,149,690 square kilometers resulting in a relatively low population density of 15 inhabitants per sq. km. The Kingdom boasts a robust economy with the highest GDP figures among other Middle Eastern nations. The development of the Covid-19 crisis in Saudi Arabia began with the official report of the first confirmed case on 2 March by the Kingdom’s Ministry of Health [9]. In less than two months, the country experienced a substantial rise in Covid-19 cases with official reports indicating that 10,484 had been infected by 20 April 2020 [9]. As the country was engulfed by the pandemic, Saudi authorities prioritized awareness programs while seeking to contain the spread of Covid-19 with the help of curfews and social distancing measures [10]. To curtail the uncontrolled spread of Covid-19 among the population, the Saudi government introduced strict limitations on social gatherings, cultural activities, and other forms of close contact. The Muslim state resorted to completely closing mosques to minimize potential human-to-human transmissions [11]. The effectiveness of Saudi Arabia’s anti-Covid-19 efforts is evidenced by the rapid decline in new cases and deaths by early summer 2020 [3]. By July, the Middle Eastern country started to revert harsh restrictions allowing citizens to engage in usual activities and even providing an opportunity for pilgrims to visit the city of Mecca.

There were no indications grounded on concrete evidence that would have suggested that statistics presented by the Saudi government are inaccurate or misleading. Although the reports on Covid-related infection rates and mortality rates are unlikely to be fabricated, it does not mean that the data provide an accurate representation of the pandemic’s development. The key concern pertains to the fact that official reports account only for confirmed cases with the latter being closely correlated with the total number of performed PCR tests [12]. By the end of summer 2020, the Saudi data on Covid-19 was obtained by performing nearly 3.3 million tests which returned a 10% positive rate [13]. As the country’s positive rate is within the boundaries set by the WHO, the confirmed cases and confirmed deaths could be reliably used by researchers. The total number of reported deaths does not raise questions as it is even higher than the global average. Another identified issue emerges as a result of the low population density and the complexity of performing testing in remote areas of Saudi Arabia, such as off-the-grid towns and communities in the Eastern Province [14]. The presence of closed-off communities with limited coverage of healthcare services is expected to skew the relationship between population density and Covid-19 statistics [9]. Importantly, the described problem is not localized within the MENA region as the logistical challenges of performing PCR tests on a global scale make it nearly impossible to assess the actual dynamics of the disease in any remote region of the world [6].

From the economic perspective, the Covid-19 pandemic both directly and indirectly disrupted a variety of Saudi Arabia’s industries and sectors. While the nine-billion-dollar budget deficit in Q1 2020 was partially tied to disturbances in the global market for petroleum products, a sizable role was attributed to the coronavirus crisis [3]. Struggling to mitigate the disastrous effects of quarantine measures, Saudi Arabia’s government stimulated the national economy by reducing spending and raising VAT. With the number of total confirmed cases and deaths approaching 360,000 and 6000, respectively, by the end of the year, Saudi Arabia incurred a budget deficit of $79 billion or nearly 300 billion SAR [14]. Considering that Covid-19 directly affects local businesses and indirectly influences global prices for crude oil, Saudi Arabia expects to reduce government expenses in 2021 to $263 billion to accommodate for the recession observed in the four quarters of 2020 [14,15].

### 2.2. The Course of the Covid-19 Pandemic in China

A country occupying the fourth largest landmass on the globe, PRC features a considerable population of 1.4 billion dispersed across sparsely populated rural regions and megacities such as Chongqing, Shanghai, and Beijing. The world’s most populated state is subdivided into twenty-eight administrative regions as well as four centrally-controlled metropolitan areas. Although the total population density is 145 citizens per sq. km, the density of urban centers such as Shanghai and Beijing is considerably higher and approaches 4200 and 1300 per sq. km, respectively. Due to the fact that the Republic of China and regions such as Hong Kong followed distinctly different approaches towards registering Covid-related incidents and mitigating economic consequences, the research focuses on PRC, with both PRC and China being used interchangeably throughout the paper. While China has been at the forefront of numerous major epidemics throughout the past millennia, the most recent incidents include the 1968 Hong Kong flu, the 1997 bird flu, and the 2003 SARS epidemic [4]. The emergence of Covid-19 is deeply tied with PRC’s Hubei province and, specifically, the city of Wuhan which is widely accepted as the location of the first outbreak. Notably, the government’s hesitance to immediately recognize the epidemic resulted in the rapid spread of Covid-19 across all Chinese territories by 29 January 2020. As the severity of the novel coronavirus infection became evident, the authorities mandated the highest response level to curb the growing number of new Covid-19 cases. With the majority of early cases being restricted to Hubei, China reported nearly 25,000 cases and 700 deaths by early February 2020 [16]. The highest peak of the pandemic coincided with the Spring Festival which led scholars to believe that population migration and population aggregation patterns played an important role in the Covid-19 spread [17,18]. Moreover, Jiang et al. noted that the distance from the epicenter in the Wuhan province emerged as one of the key determinants for the epidemiological situation in Chinese provinces [19]. In the wake of the Covid-19 pandemic, PRC introduced increasingly strict social distancing measures as China’s government enforced a strict quarantine in all provinces. Thanks to city-wide lockdowns and movement restrictions, China successfully contained the pandemic on its territories by March 2020 [4]. At the time of the study, the total number of confirmed cases and deaths was estimated at 86,000 and 4600, respectively.

However, the veracity of data and accuracy of reports presented by China’s Communist Party had been the subject of critique for the entire duration of the pandemic. Scholars and governments questioned not only China’s methods of containing the pandemic but also the validity of information on Covid-related morbidity and mortality [20,21]. The non-democratic country has been accused of publishing imprecise and even fabricated statistical data to preserve its image on domestic and international scenes. Despite the access to a robust economic base and nearly absolute control over media resources and industries, PRC was unable to conceal the existence of the problem. If not being completely truthful, the statistical records published by the government are expected to be somewhat accurate in depicting the proportional distribution of cases and deaths across regions [21]. Furthermore, the unfortunate incidents of suppressing whistleblowers in the healthcare system and authoritarian policies could have become the reason for data manipulations at the sub-national level [22]. As these manipulative reports by local governments consistently appeared in early 2020, PRC’s Premier of the State Council Li Keqiang indirectly confirmed suspicions of the international community [20]. Despite the apparent lack of integrity and insufficient accuracy of Chinese official statistics on Covid-19, the problem is not limited to this Asian country. In fact, evidence of manipulation with official reports on Covid-19 morbidity and mortality can be found in most democratic countries such as the US and Germany [23]. Although there is a clear indication that global statistics regarding the spread and severity of the coronavirus pandemic is not entirely accurate at best and misleading at worst, the current scholarly consensus is to continue with caution when relying on governmental reports due to the possibility of manipulations [24]. 

Although PRC’s anticipated economic growth in 2020 was estimated to be at 5.9%, Covid-19 had profound effects on all sectors including the transportation industry, tourism, banking, and others [1]. For example, the efforts to limit interprovincial movement led to the reduction of total train trips by 73% as compared to previous years and a considerable drop in sales of motor vehicles. During the period of harshest quarantine measures, China mandated the closure of businesses, industries, restaurants, and educational institutions [2]. Similar to Saudi Arabia’s response to Covid-19, PRC mandated a near-complete shutdown of the tourism industry and curtailed all commercial flights in the country. Among the most controversial yet notable events was the extensive ban on the wildlife trade sector which reportedly amounted to more than $74 billion [25]. The singular focus on wildlife trade has both negative and positive economic connotations but most scholars recognize this decision as an effective response to the global pandemic [26,27]. In this context, international observers had estimated that PRC’s economic growth would shrink to less than 2.3% in 2020 [28]. The timely implementation of anti-pandemic measures helped to alleviate the economic downturn in China as the expected growth for 2021 is likely to reach 8.4% [28].

### 2.3. Population Density and Covid-19

Considering the decisive role of human-to-human transmission for spreading the coronavirus disease, population density emerges as one of the most apparent factors influencing infection rates. From this perspective, numerous experts emphasize that people living in densely populated areas are more susceptible to being infected as opposed to the inhabitants of low-density rural regions [29,30]. The proponents of this theoretical approach indicate that countries with high population density, such as India and China, are likely to experience surges in Covid-19 cases and deaths. Several empirical inquiries have been made in Italy and Brazil regarding the potential feasibility of using population density as a determinant for Covid-19 spreading in different regions [31,32]. Although the opposing viewpoint on the issue will be discussed below, population density emerges as a crucial anthropogenic factor because the increasing frequency of interactions between people naturally contributes to high infection rates. Several authors provided plausible evidence pointing to the positive causative relationship between population density and the incidence of infectious diseases such as Covid-19 [33]. With people being more likely to engage in direct contact with others in crowded areas, the evidence further suggests that overpopulated cities and districts are prone to comparatively high infection rates [17,19]. Most authors agree that high-density cities provide conditions for crowding which offers an additional venue for viral transmission. 

Despite the extensive empirical evidence indicating the significance of population density as a determinant of Covid-19 spread, the findings vary from country to country and from region to region. The pandemic lasted for less than 12 months by the time of this study but a comprehensive body of knowledge started to form with regard to the interrelation of socio-demographic factors and lockdown policies [34]. From this perspective, countries that implemented strict lockdown policies were expected to report lower infection rates and mortality rates in highly populated regions. As strict lockdown policies are especially effective in densely populated areas with expansive state control over the population, non-democratic nations showed the weakest causal relationships between the parameters investigates within the scope of the present research [35]. The described assumption does not deviate from the aforementioned claims because population density can still be used as a predicting factor during the early stages of an infectious outbreak as had been outlined in past studies. Moreover, countries with comparatively weak state control and disobedience among citizenry are those associated with the strongest positive correlation between population density and Covid-19 spread [32]. The strengths of causal relationships further vary across different cities and townships in the same country with the majority of reports confirming that low population density is tied to low infection rates [36]. There is no conclusive data on the relationship between population density and Covid-19 morbidity in sparsely populated regions. 

Distinctly from the theory outlined above, certain researchers revealed that the Covid-19 pandemic does not always follow the expected pattern of high infection rates in densely populated regions. For example, Hamidi et al. [5] performed a meticulous analysis of Covid-19 cases and deaths in American counties with different population densities. While the authors concluded that density has no meaningful relationship with Covid-19 infection rates, high-density areas are considerably more likely to be associated with lower mortality rates. Several other authors confirm the described observation by explaining that people living in urbanized regions are better informed regarding social distancing measures and have access to superior health care than their rural-dwelling counterparts [37,38]. Additionally, skeptics note that metropolitan size and transportation links emerge as more significant determinants of Covid-19 infection rates and deaths than population density [39]. The skepticism is shared by scholars who accounted for lockdown policies because population density does not appear to be a significant determinant of Covid-19 incidence in some of the most densely populated countries of the world [33]. While multiple studies focusing on China provided inconclusive results, the authors recommended further investigation of the issue [35,40]. No such studies seeking to connect population density and Covid-19 mortality and morbidity were performed in Saudi Arabia to this day. The disagreement among scholars on the role played by population density in the development of pandemics is one of the principal motivators for the current research. Due to the inconclusive empirical data on the topic, it is crucial to investigate the practical significance of population density as a causative factor for newly emerging infectious diseases such as Covid-19 [41]. Previous studies emphasized the need of uncovering to what extent population density correlates with epidemiological variables such as infectivity rates and mortality rates in countries with strict lockdown policies. In this framework, the study seeks to assess the role of population density as a predictor of Covid-19 cases and deaths in Saudi Arabia and China after the peak of the Covid-19 pandemic in these two countries. 

### 2.4. Kissinger’s Malthusian Theory and Future Pandemics

Declassified in the 1990s, Henry Kissinger’s 1974 report touched on the problem of worldwide population growth as a possible threat to the national interests of the US. The key idea of the document was that unchecked population growth in underdeveloped nations puts a strain on the global supply of food, minerals, and other resources. Whether due to possible civil disturbances or overconsumption of resources, the report recommended preemptively addressing the uncontrollable population growth by implementing population reduction strategies focused on promoting abortion, contraception, and family planning [42]. After being declassified, Kissinger’s Malthusian theory was met with mixed responses from scholars who either proposed new Neo-Malthusian models or discarded the theory as unethical. With the recent addition of the global pandemic, the growing importance of social issues, famine, and environmental concerns revitalized the interest in population reduction as a viable long-term solution [43]. The recent examples of applying disincentives and coercion techniques informed by Neo-Malthusian models were reported in China and India. In line with China’s one-child and two-child policies, there are multiple reports of coerced abortions, coerced sterilization, and country-wide propaganda efforts intended to control population growth [44]. While there are no such policies in India, authorities consistently pushed population reduction measures by indirectly penalizing families with more than two children [45]. Such methods were not widely used or considered for use in Saudi Arabia. Overall, the viability of Neo-Malthusian models to alleviate future pandemics remains understudied with nearly no data being available in regard to Covid-19.

The ongoing pandemic exacerbated socio-economic challenges encountered by the populations of rapidly developing non-democratic countries such as China and Saudi Arabia. As both China and Saudi Arabia enacted strict lockdown measures, less affluent citizens lost access to critical services and products which contributed to the already-existing lack of food security. Unable to survive without stable income, foreign workers and poor citizens experienced the devastating social impacts of an epidemiological emergency first-hand [46,47]. In this context, the Covid-19 pandemic showcased the need for robust private and public policies aimed at achieving food security and food sustainability. Although the governments of China and Saudi Arabia sought to prioritize health security, the lockdown measures were implemented at the expense of the least protected groups living in densely-populated regions. The absence of effective strategies for ensuring food provisioning and food security during health crises emerges as the critical concern for all nations who need to develop resilient systems for supplying and procuring food to the affected population [48]. In this framework, Neo-Malthusian models could provide a solution by informing comprehensive policies that would help to reduce population density and mitigate future medical disasters. Taking into account that the continuous population growth in Chinese territories is likely to be unsustainable from the long-term perspective, population density should be approached as one of the indicators guiding the gradual introduction of population reduction policies and initiatives [49]. Finally, the Covid-19 crisis highlighted the need for public programs that would not only address overpopulation but also tackle associated social concerns such as food security and sustainability [50].

With high infectivity and mortality being arguably a definitive characteristic of overpopulated regions, the authors propose that a Neo-Malthusian model informed by Henry Kissinger’s population reduction theory can be applied as a control measure to control future pandemics [51]. In this framework, sparsely populated countries and regions can also benefit from policies informed by different population reduction frameworks. As the world’s population will inevitably increase in the upcoming decades, the majority of regions that are currently sparsely populated are likely to be densely populated by the end of the twenty-first century [42]. Expected to surpass density levels at which pandemics can be effectively managed with lenient lockdown policies, many countries would have to either resort to strict lockdown policies, such as China’s, or adopt a Neo-Malthusian model [21]. However, the viability of Neo-Malthusian models depends on a government’s ability to manage its citizenry and implement potentially non-democratic policies. If the investigation uncovers that high-density Chinese regions are more susceptible to Covid-19 than low-density Saudi cities, Kissinger’s Malthusian theory and Neo-Malthusian models can be used as a basis for reducing the impacts of future pandemics on the economic growth in the two countries. On the other hand, the absence of a causal relationship between population density and Covid-related morbidity and mortality in the studied countries would not invalidate the assumption that population reduction can be a viable solution for epidemiological, environmental, and social concerns. Within the scope of the described theoretical tenets, the study starts by assessing the role of population density as a predictor of Covid-19 cases and deaths in Saudi Arabia and China during the Covid-19 pandemic. Consequently, the investigation shifts towards the possibility of addressing the current and future epidemiological crises with the help of Neo-Malthusian theories. As a result, the current empirical study is informed by the following research question: What are the merits of using Neo-Malthusian models for reducing the impacts of pandemics on the economic growth in countries with low population density?

## 3. Methodology

Under the scope of the current study, the principal goal is to investigate the possibility of a correlational relationship between population density in Chinese and Saudi Arabian regions and Covid-related cases of infections and deaths. If the authors confirm the existence of correlation as was theorized, the consequent stages would pertain to modeling the linear relationship between population density and Covid-related deaths and cases as an independent variable and dependent variables, respectively. With infection rates and mortality being perceived as a possible function of population density, the authors consider the latter’s significance for economic growth in the selected countries. Furthermore, the economic significance of the pandemic is analyzed through the lens of Kissinger’s Malthusian theory. At this stage, there is no cohesive theoretical framework for describing the spread of highly infectious viral diseases in areas with different population densities and their impact on economic growth. Moreover, conflicting views on the topic necessitate additional research presenting a comparative analysis of high-density Chinese regions and low-density Saudi regions. 

### 3.1. Data Used

Considering dependent variables for the study, the authors collected data for both infection cases and deaths caused by Covid-19 per 100,000 people for both countries with regions being selected as a unit of analysis. Furthermore, the research relies on the up-to-date dataset containing cumulative confirmed cases and mortalities for each province until 4 January 2021. Although the pandemic started and reached its peak at different timeframes in the two countries, the researchers are interested in comparing the results after the pandemic has been contained to a reasonable degree which justifies the choice of cumulative records for daily infection rates/mortality rates. Different reference dates would have no impact on the results because the study is focused on static data accumulated throughout a prolonged period as opposed to dynamic records. In the case of China, the dataset for 30 provinces, municipal areas, and territories was extracted from the JHU Covid-19 Resource Center [52]. To ensure normality, reports concerning Hubei were excluded due to the disproportionately high rates of infectivity and mortality as compared to other administrative units of PRC. On the other hand, the dataset for 13 provinces of Saudi Arabia was collected from the Covid-19 Dashboard operated by Saudi Arabia’s MOH [53]. Both datasets contained full and up-to-date information pertaining to Covid-related deaths and Covid-19 cases in China and Saudi Arabia. 

As the population density was chosen as the independent variable for the study, the authors calculated the demographic characteristic for each province by dividing the total number of residents by the land area of the respective province. With the province being the unit of analysis for the research, the population density represented with the number of citizens per sq. km was identified for China’s 30 provinces and Saudi Arabia’s 13 provinces. Specifically, the authors extracted the information regarding the total population for Chinese regions from the National Bureau of Statistics of China’s database, with figures being the estimates for 2017 based on national sample surveys [54]. From the General Authority for Statistics’ Demographic Survey, the authors obtained population figures for all Saudi provinces in 2016 [55]. The information from all databases for both countries was extracted and labeled for the consequent use with the R statistical software for correlation analysis and linear regression.

The author’s primary goal is to identify a possible correlation between population density and Covid-related infection cases as well as mortality in administrative units of China and Saudi Arabia. After evaluating the existence and strength of correlation between variables, the following step includes modeling of simple linear regression data plots visualizing the linear relationship between the following variables: population density and Covid-19 cases in China; population density and Covid-19 mortality in China; population density and Covid-19 cases in Saudi Arabia, and; population density and Covid-19 mortality in Saudi Arabia. The obtained results are further reviewed and discussed through the lens of Kissinger’s Malthusianism theory and its importance for economic growth in low-density and high-density regions.

### 3.2. Hierarchical Cluster Method

We employed the hierarchical cluster method to facilitate the comparison of Saudi and Chinese provinces based on the infection rates and population density. The average-linkage approach to the hierarchical agglomerative cluster analysis was deemed suitable for grouping distinct administrative regions for each of the investigated countries. The chosen technique helps to identify the groups of provinces with highest and lowest infection rates with regard to their respective population density figures [56]. The two-step clustering process pertained to the consecutive grouping of provinces based on the two dependent variables; consequently, we created clusters that would further incorporate the data on population density.

### 3.3. Pearson’s Correlation

The authors calculate Pearson’s two-tailed coefficient and R Square to establish nature as well as the strength of linear relationships between the two dependent variables and the independent variable. The computation is performed with the help of the standard formula for the product-moment correlation coefficient by separately using the datasets for China and Saudi Arabia. Furthermore, the null hypothesis test allows confirming the existence of a linear relationship in regard to the selected variables. After comparing the variance of variables with the help of Fisher’s transformation and establishing the standard error, it is also possible to calculate the *p*-value for each pair of variables [57]. For the purposes of the study, *p* < 0.05 is the threshold of statistical significance. The formula used for the correlation equation is presented below.

(1) $$r=\sum \left(x-mx\right)\left(y-my\right)÷\sqrt{\sum \left(x-mx\right){}^{2}\sum \left(y-my\right){}^{2}}.$$

Apart from the main data set including all provinces and administrative regions of China and Saudi Arabia, correlation coefficients were additionally calculated for a separate model based on the average population density within both countries. In Saudi Arabia, population density is higher than the country-wide average of 15.6 people per sq. km in the following territories: Al Bahah, Al Qaseem, Jazan, Aseer, Ar Riyad, and Makkah al Mukarramah. The remaining geographic regions correspond to the vast swaths of sparsely inhabited land such as the Rub’ al-Khali dessert. The median density for the group of densely populated territories is 38.4 per sq. km, and for the group of sparsely populated it is 6.2 per sq. km. As for PRC, the Heihe-Tengchong Line was employed as an accepted approach for demarcating highly-populated and sparsely-populated provinces in China. To ensure consistency, we also included Yunnan and Jilin to the following list of regions with population density being below the country average of 148 people per sq. km: Tibet, Qinghai, Xinjiang, Inner Mongolia, Gansu, Heilongjiang, and Ningxia. The median density for the group of sparsely populated territories to the West of the Heihe-Tengchong Line is 57.41 per sq. km, and for the group of densely populated it is 373.2 per sq. km.

### 3.4. Simple Linear Regression

Simple linear regression is performed to understand the underlying relationships between the chosen sets of variables for both countries. With the help of the regression line based on the standard formula for the method of least squares, the authors seek to illustrate the significance of population density as a determinant of Covid-19 cases and mortality over the observed period. The following mathematical equation underlies the process of modeling simple linear regression:

(2) $$y=a+bx,\;\mathrm{with}\;b=\sum \left(x-\bar{x}\right)\left(y-\bar{y}\right)÷\sum \left(x-\bar{x}\right){}^{2}.$$

Moreover, the simple linear regression provides an opportunity to model the aforementioned relationship with the intention of better understanding the role of the independent variable. To preserve the integrity of results, the authors ensure the following characteristics: normality, linearity, homoscedasticity, and independence of observations [57]. For the purposes of data analysis and computations, the R statistics software has been employed by the authors to process the extracted datasets for China and Saudi Arabia.

### 3.5. Conceptual Framework 

The authors rely on empirical analysis of primary and secondary data to explore the possibility of applying Neo-Malthusian models to alleviate the detrimental impacts of future pandemics. Due to the absence of scholarly consensus on the role of population density as a determinant of Covid-19 morbidity and mortality, the present research performs a comparative analysis focusing on China and Saudi Arabia, two non-democratic states that have implemented strict lockdown policies to curb the pandemic. In this study, pragmatism was the chosen philosophical paradigm that helped to structure the scholarly inquiry in regard to the data collection and analysis for the purposes of answering the posed research question. The grounded theory underlies the comparative analysis by considering both primary and secondary data to investigate the studied problem and reach a plausible solution. The authors performed a comparative analysis of Saudi Arabia and China while using the resulting findings as a basis for exploring the possibility of applying Neo-Malthusian models for addressing future pandemics. With Saudi Arabia being sparsely populated and not engaged in population reduction programs, China emerges as a densely populated country that continuously relies on policies informed by Neo-Malthusianism. Throughout the 1970s, numerous scholars revisited the ideas proposed by Malthus in his 1798 essay [58,59]. Culminating with Kissinger’s report, these studies concluded that current growth rates are unsustainable from the perspectives of food supply, environmental stress, and the availability of natural resources [45]. In this framework, a model informed by Neo-Malthusian principles could be used for the purposes of long-term population control in countries susceptible to these issues. Drawing from the aforementioned theories, the authors seek to analyze the viability of Neo-Malthusian models for minimizing the detrimental impacts of pandemics such as the Covid-19 pandemic.

## 4. Results and Discussion

By the time of the research, Saudi MOH reported the following cumulative figures from the pandemic’s onset: 363,259 Covid-19 cases and 6265 deaths. In contrast, PRC confirmed 97,028 cumulative cases of Covid-19 infection and 4792 deaths by the same date. As China’s population at 1.3 billion in 2017 is substantially larger than that of Saudi Arabia with 31.8 million inhabitants, the Covid-related figures were converted to represent incidence rates per 100,000 people. In the case of Saudi Arabia, 13 emirates substantially differ in population density as well as the number of reported cases and deaths, with Jazan being the most densely populated region of the country. On the other hand, among China’s thirty administrative units selected for the research, the following cities feature the highest density: Shanghai, Tianjin, and Beijing. 

**Figure 1:**
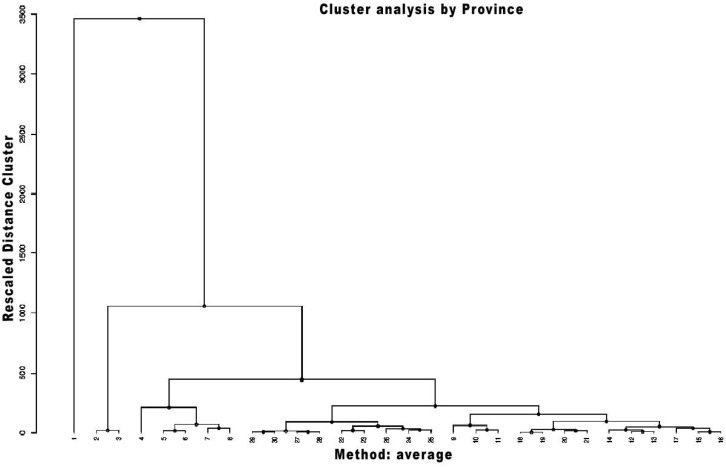
Cluster analysis of population density and Covid-19 cases for China’s administrative regions.

**Figure 2:**
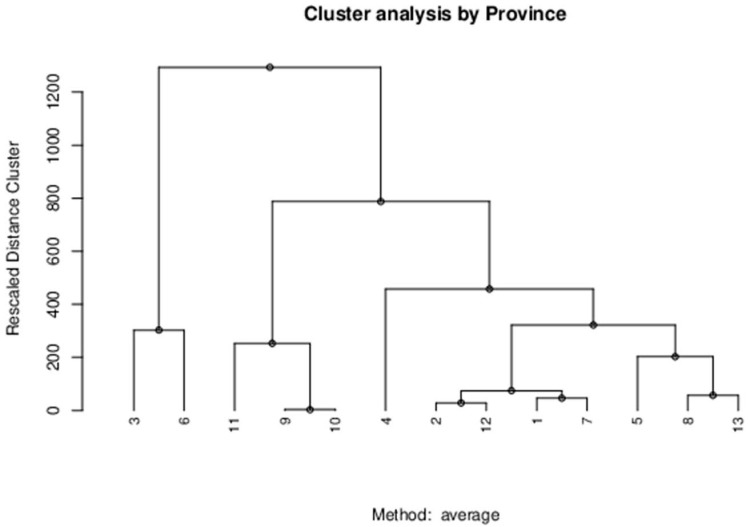
Cluster analysis of population density and Covid-19 cases for Saudi Arabia’s administrative regions.

By employing the hierarchical agglomerative cluster method based on the average-linkage approach, we initially classified the regions of Saudi Arabia and China based on the infection rates. The division into groups facilitated the consequent clustering process based on the population density in each of the aforementioned regions. The study population of 30 administrative regions in China was divided into groups as displayed on the dendrogram (see Figure 1). The first group includes Shanghai with estimated population density of 3814 inhabitants per sq. km and Covid-19 cases of 6.32 per 100,000. Tianjin and Beijing comprise the second group while the remaining regions form several smaller clusters corresponding to their estimated population density and Covid-19 cases. In the case of Saudi Arabia, 13 emirates were divided into multiple groups that included Eastern Province and Al Madinah Al Munawwarah, Ha’il and Najran, and others (see Figure 2). The hierarchical clustering did not reveal any significant insights after classifying the emirates by the population density and Covi-19 cases. 

**Figure 3:**
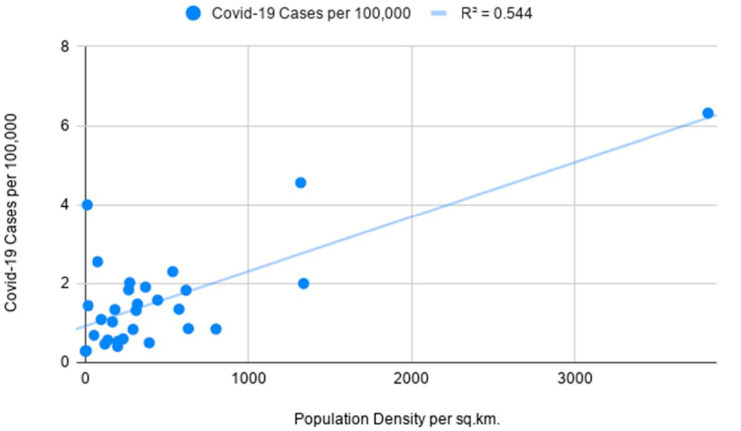
Simple linear regression with population density as an independent variable and Covid-19 cases as a dependent variable for China’s administrative divisions.

**Figure 4:**
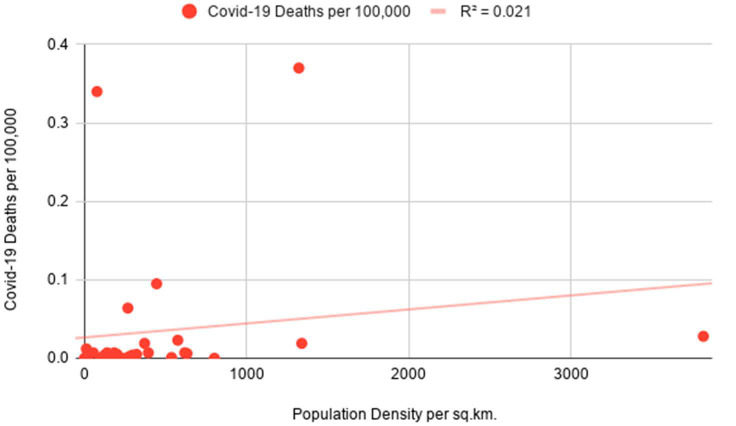
Simple linear regression with population density as an independent variable and Covid-19 deaths as a dependent variable for China’s administrative divisions.

The preliminary analysis of data strongly suggests that Covid-19 infections in predominantly sparsely populated Saudi Arabia are not representative of population density figures. When excluding Hubei from the analysis, the opposite is evident in the case of China with high density being closely tied to the rise in Covid infections as seen in Figure 3. Notably, the mortality rate from Covid-19 appears to be higher in high-density areas of Saudi Arabia as compared to the lack of a meaningful connection in Chinese regions as depicted in Figure 4.

**Figure 5:**
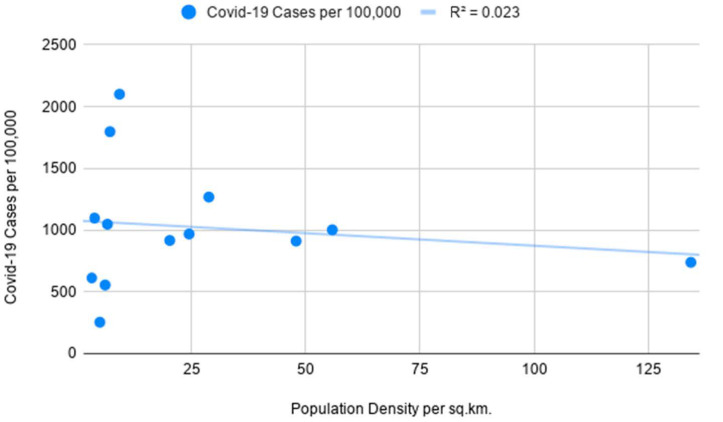
Simple linear regression with population density as an independent variable and Covid-19 cases as a dependent variable for Saudi Arabia’s administrative divisions.

**Figure 6:**
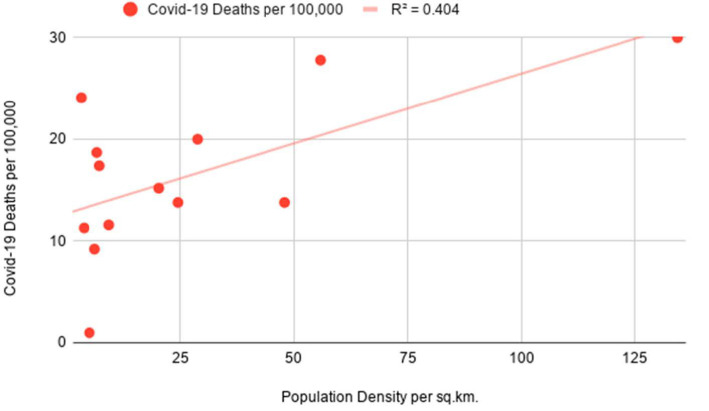
Simple linear regression with population density as an independent variable and Covid-19 deaths as a dependent variable for Saudi Arabia’s administrative divisions.

The following correlation coefficients are computed for independent and dependent variables for China’s administrative regions: 0.73 for Covid-19 cases indicating a moderate positive correlation and 0.14 for Covid-19 deaths indicating a weak positive correlation. Furthermore, the coefficients for Saudi Arabia are as follows: −0.15 for Covid-19 cases indicating a weak negative correlation and 0.63 for Covid-19 deaths indicating a moderate positive correlation which is depicted in Figure 5. In the course of the significance test, the authors calculated *p*-values for China’s Covid-19 infection rates and Saudi Arabia’s mortality rates to be smaller than 0.05 at the chosen significance level, allowing us to reject the null hypothesis. As the *p*-value for China’s mortality rates is 0.44 and the *p*-value for Saudi Arabia’s infection rates is 0.64, the null hypothesis cannot be rejected and results should be considered not significant as *p* is larger than 0.05. As evident in Figure 6, the linear regression model for Saudi Arabia presents counter-intuitive results with a slight indication that higher population density could be a minor determinant for mortality rates as a result of Covid-19. The number of Covid-related deaths per 100,000 people in Saudi Arabia is not homogenous across all the regions. The empirical analysis shows that it is consistently higher in densely populated emirates as compared to sparsely populated ones. The latter is evidenced by Covid-19 mortality rates in the most overpopulated regions of the country—Jazan and Makkah Al Mukarramah. Interestingly, sparsely populated Eastern Province and Northern Borders reported unusually high mortality rates which can be explained by inconsistent diagnostic approaches in Saudi medical centers. While we do not have information on how Covid-related deaths were recorded in these emirates, the number of medical institutions could have been another determinant. Only in the case of China’s infectivity rates, the obtained results align with a presumption that highly-dense areas are more prone to the spread of infectious diseases.

At the R Square values of 0.54 (for China’s infection rates) and 0.4 (for Saudi Arabia’s mortality rates), the results indicate that the independent variable can partially explain the incidence of Covid-19 cases in China and the mortality rates in Saudi Arabia, respectively (Table 1). Low R Square values for China’s infection rates (0.02) and Saudi Arabia’s mortality rates (0.02) signify that the independent variable is not responsible for the majority of variations of the studied dependent variables. The lack of definitive and strong relationships between studied variables can be explained by the fact that population density is one of the many factors influencing the course of infectious diseases in different regions. Furthermore, the higher number of recorded Covid-19 deaths in Mecca and Jazan is possibly tied to superior diagnostic methods available in the metropolitan areas of the two regions. While Covid-related mortality rates in all Chinese regions are extremely low, the regression model for population density and infection cases aligns with the theory that the former can act as one of the determining factors for the latter. After separately assessing the data for densely-populated and sparsely-populated territories in both countries, the results proved to be largely not significant at *p* < 0.05. The sole exception pertained to Covid-19 cases in Chinese provinces to the east of the Heihe-Tengchong Line which indicated a relatively strong positive correlation with significant results at *p* < 0.05. The assessment of R Square values does not show considerable differences after separately analyzing the data for regions with different population density averages.

As evidenced by the results, the chosen methodology and data are prone to certain limitations that include the rapid development of the Covid-19 situation in different countries, the lack of conclusive data, and a non-uniform approach to recording and reporting Covid-related events. In this framework, a longitude time-series research would be uniquely suited to further explore the topic and provide robust evidence regarding the role of population density as a determinant of Covid-19 infection and mortality rates. Moreover, as the pandemic peaked at different periods in Saudi Arabia and China, the two countries relied on dramatically different approaches for reporting new cases, treating Covid-19 patients, and implementing anti-pandemic measures. From this perspective, future studies should account for distinctions in health systems as well as for numerous confounding factors such as pre-existing medical conditions.

The data analysis indicated that population density has no significant causal relationship with Covid-19 morbidity and mortality which suggests that other factors play a more significant role concerning the Covid-19 spread. The effectiveness of lockdown policies implemented by Saudi Arabia and China, two non-democratic regimes, may obscure the actual connection between the studied variables. In this framework, the existing evidence is not sufficient to conclude whether Covid-19 is a Malthusian event or not. Nevertheless, the concepts developed by Malthus and further explored in Kissinger’s report may inform the solution to the ongoing environmental, social, and epidemiological crises. As the population growth puts a strain on the global supply of resources, this includes the ability of countries to provide healthcare services to the ever-increasing population. By considering various population reduction strategies, governments would be able to align with the requirement of sustainable development. Despite the fact that Neo-Malthusianism remains a highly controversial theory, its implementation can indirectly contribute to the resolution of pertinent problems such as food shortages and epidemics [60]. The latter is evident in the case of China which has been arguably effective in addressing the Covid-19 outbreak while also practicing several population control measures [16,17,21]. Finally, the authoritarian qualities of both governments presented an opportunity to maintain strict lockdown policies which suggest the potential for adopting a form of Neo-Malthusianism to preserve China’s and Saudi Arabia’s economic growth in the aftermath of the pandemic. Population density can be considered as one of the numerous determinants associated with health crises such as the Covid-19 pandemic. Taking into account socio-economic threats and epidemiological concerns arising as a result of overpopulation, a combined work of scholars and professionals is necessary to propose effective Neo-Malthusian models that would act as a long-term solution for future disasters and health crises.

Considering that the research did not provide uniform evidence that population density has a strong positive relationship with Covid-19 cases and deaths, population control informed by Neo-Malthusian models is likely to be rather ineffective as a direct Covid control measure. As seen in the example of densely populated regions of China, the ensuing economic recovery was primarily tied to centrally-planned policies and effective quarantine measures implemented by the government. From another perspective, Covid-19 events in low-density regions of Saudi Arabia are preeminently tied to other confounding factors that should be further explored in future studies. Furthermore, both global prices for crude oil and Covid-19 are responsible for Saudi Arabia’s economic decline in 2020. As China and Saudi Arabia managed to achieve meaningful economic recovery after containing the coronavirus, Kissinger’s Malthusian theory of population reduction is likely to be ineffective as a control measure for future pandemics. Population reduction approaches should be further explored after identifying other major determinants for Covid-19 infection and mortality along with population density.

## 5. Conclusions

The Covid-19 pandemic emerged as the most significant crisis of the twenty-first century that continues challenging health experts and economists across the world to find suitable solutions to the ever-increasing number of epidemiological, social, and environmental concerns. Due to the infection’s rapid spread across the globe, the existing evidence and knowledge base are constrained by the lack of data. In this context, the pandemic’s economic impact remains largely understudied as many potential determinants of Covid-19 infection and mortality rates remain hidden. The role of population density as a factor contributing to the course of an emerging infectious disease is somewhat controversial with several authors presenting contrasting viewpoints on the topic. The current study did not uncover a meaningful and conclusive causal relationship between the studied variables suggesting that population density does not explain variations in Covid-related mortality and infection rates. Although Covid-19 does not appear to be a Malthusian event, the possibility of using Neo-Malthusian models to address future pandemics should be explored further. Furthermore, the findings indicate that the role of population density could be obscured by other factors such as statistical inaccuracies and lockdown policies. There is a need for additional studies on the importance of strict lockdown policies in non-democratic nations as an effective anti-pandemic measure. In the case of population control, Neo-Malthusianism remains a viable solution to the problems posed by the uncontrolled population growth that should be further investigated. Although a moderate positive correlation was observed between the independent variable and China’s infection rates and Saudi Arabia’s mortality rates, the results are inconclusive due to the potential impact of other factors. In the case of China and Saudi Arabia, the analysis of Covid-19 cases and deaths in comparison to the population density of different regions did not reveal any conclusive evidence that population control can be used as a Covid control measure. However, the analysis indicates that Neo-Malthusian models could be considered by non-democratic governments to implement long-term policies that would facilitate economic recovery in the post-pandemic period. The research reveals that the severity of health crises such as the Covid-19 pandemic is closely tied to an array of social and economic problems. To ensure food security and sustainability consistently threatened by overpopulation, the authors recommend to prioritize scholarly investigations of Neo-Malthusian models as possible solutions intended to mitigate future disasters and health crises. Future research is necessary to investigate population control policies as a possible approach to alleviating the economic impact of global pandemics.

## Figures and Tables

**Table 1:** Coefficients of determination and *p*-values.

		Saudi Arabia		China	
		Cases	Deaths	Cases	Death
All territories	R Square	0.02	0.4	0.54	0.02
	*p*-value	0.62	0.01	<0.00001	0.44
Densely populated regions	R Square *p*-value	0.41 0.16	0.6 0.06	0.74 <0.00001	0.05 0.32
Sparsely populated regions	R Square *p*-value	0.53 0.06	0.07 0.55	0.04 0.58	0.01 0.73

## Data Availability

The datasets pertaining to Covid-19 cases and mortality used in the current study were obtained from Saudi Arabia’s MOH (https://covid19.moh.gov.sa/, accessed on 6 January 2021) and JHU Covid-19 Resource Center (https://coronavirus.jhu.edu/data/new-cases, accessed on 6 January 2021). Other demographic datasets were extracted from China’s Bureau of Statistics (https://data.stats.gov.cn/, accessed on 6 January 2021) and Saudi Arabia’s General Authority for Statistics (https://www.stats.gov.sa/, accessed on 6 January 2021).
